# Diagnostic ambiguity of aseptic necrobiosis of a uterine fibroid in a term pregnancy: a case report

**DOI:** 10.1186/s12884-018-2154-x

**Published:** 2019-01-07

**Authors:** Julius Sama Dohbit, Esther Ngo Um Meka, Joel Noutakdie Tochie, Igor Kamla, Celestin Danwang, Frank-Leonel Tianyi, Pascal Foumane, Gervais Ondobo Andze

**Affiliations:** 1 Departement of Gynaecology and Obstetrics, Yaounde Gynaeco-Obstetrics and Paediatric Hospital, Yaoundé, Cameroon; 2 Departement of Gynaecology and Obstetrics, Faculty of Medicine and Biomedical Sciences, University of Yaoundé 1, Yaoundé, Cameroon; 3 Departement of Surgery and Specialities, Faculty of Medicine and Biomedical Sciences, University of Yaoundé 1, Yaoundé, Cameroon; 4 Department of General Medicine, Mayo Darle Sub-Divisional Hospital, Banyo, Cameroon; 5 Departement of Surgery, Yaounde Gynaeco-Obstetrics and Paediatric Hospital, Yaoundé, Cameroon

**Keywords:** Uterine fibroid, Leiomyoma, Pregnancy, Aseptic necrobiosis, Red degeneration

## Abstract

**Background:**

Uterine fibroids are the most common uterine tumours in females of reproductive age. During pregnancy, uterine fibroids may be complicated by aseptic necrobiosis. We herein report an ambiguous clinical presentation of uterine fibroids in pregnancy and discuss the diagnostic challenges encountered in our resource-constraint setting.

**Case presentation:**

A term pregnant Cameroonian woman was admitted to our maternity unit with clinical findings suggestive of a strangulated umbilical hernia. She underwent an emergency caesarean section which fortuitously revealed aseptic necrobiosis of a uterine fibroid, managed within the same surgical intervention by myomectomy. Her post-operative course was uneventful.

**Conclusion:**

The authors highlight the need for a high index of suspicion by healthcare providers, as well as the need for a multidisciplinary approach for a favourable maternal and foetal outcome.

## Background

Leiomyomas (uterine fibroids) are the most common benign tumors of the uterus [1]. They are very common, affecting 40% of women of reproductive age and their frequency increases with age, from a prevalence of 50% at age 35 years, to 80% at age 50 years [1]. An increase in the age of first pregnancy from 24 years in 1978 to 30 years in 2012 has seen an increase in the occurrence of uterine fibroids in pregnancy [2, 3]. Pregnant women with uterine fibroids are at an increased risk for complications such as; miscarriages, preterm labour, malpresentation, labor dystocia, cesarean sections and postpartum haemorrhage [4, 5]. Uterine fibroids often increase in size during pregnancy, this increases their risk for complications such as ‘red degeneration’ or aseptic necrobiosis [5] and leads to frequent diagnostic ambiguity of obstetrical or gynaecological pathologies in resource-challenge settings [6]. Herein, we report the case of an aseptic necrobiosis of a uterine fibroid mimicking a strangulated umbilical hernia on a term pregnancy.

## Case presentation

A 35-year-old G_2_P_0010_ Cameroonian student at 39-weeks pregnancy was referred to the surgical unit of the Yaounde Gynaeco-Obstetrics and Paediatric Hospital for the management of a strangulated umbilical hernia. She had a sudden onset of localized umbilical pain three hours prior to consultation. The pain was of moderate intensity, crampy in character, aggravated by walking, without any change in bowel movement and no vomiting. An abdominal ultrasound scan revealed a parietal defect of the umbilicus measuring 55 mm in diameter with a poorly vascularised hypoechoic mass (doppler scan) measuring 50 × 30 × 37 mm, 29.6 ml in volume. In addition, the foetus was viable with a normal biophysical score and a good concordance between clinical and sonographic dating of gestational age. Hence, she was referred for surgical management of a strangulated umbilical hernia in a term pregnancy.

An episode of severe malaria during her previous pregnancy was at the origin of a spontaneous abortion at 10 weeks of gestation. Her current pregnancy was being followed at the Efoulan District hospital in Yaounde where she had attended six antenatal clinics. A urine dipstick at 24 weeks of gestation revealed a proteinuria of 600 mg/l coupled with an increase blood pressure to 152/98 mmHg and the development of lower limb oedema. She was diagnosed with pre-ecclampsia and placed on alphamethyldopa 250 mg twice daily. A second trimester ultrasound revealed the presence of two anterior and posterior interstitial myomatous nuclei, of 51 mm and 73 mm long axis respectively.

**Fig. 1:**
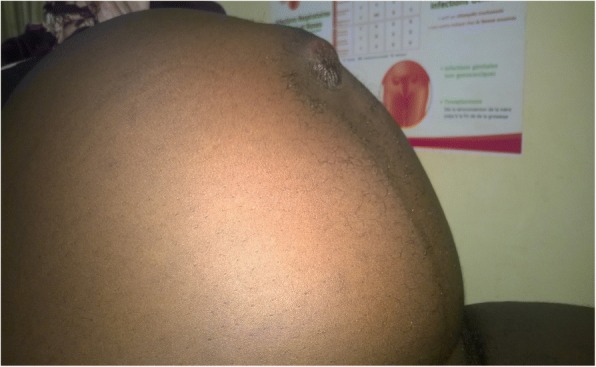
Umbilical swelling

On physical examination, the patient was in severe pain (visual analogue scale of 9/10 cm) with a temperature of 38.1 °C, pulse rate of 112 beats per minutes, respiratory rate of 22 breaths per minutes and blood pressure of 170/118 mmHg. Abdominal examination showed a gravid uterus with a uterine fundal height of 38 cm. There was a tender, non-reducible umbilical swelling (Fig. 1), with no cough impulse. There was no sign of peritoneal irritation. She had no costovertebral angle tenderness. Bowel sounds were present and normal. Her digital rectal examination was unremarkable. The foetus had a longitudinal lie, cephalic presentation, right-occipito anterior position and a fetal heart rate of 140 beats per minute. On vaginal examination, the cervix was posterior, non-effaced and closed. She had a bilateral pitting lower limb oedema extending to both knees. In view of this clinical picture, we thought of a strangulated umbilical hernia. All of these on a probable background of severe pre-eclampsia. The laboratory panel requested on admission is illustrated in Table 1.

**Table 1:** Summary of essential laboratory investigations

Laboratory test	Values	Reference values
Proteinuria	1000	< 300 mg /l
Urea	0.28	0.5–0.45 g/l
Creatinaemia	7.8	6–13 mg/l
White blood cell count (WBC)	8660	4000–10,000/mm^3^
Haemoglobin	10.4	12-15 g/dl
Haematocrit	31.4	35–49%
Prothromine Time (PT)	92	70–100%
Cephalokaoline time (TCA)	31	28–33 s
Alanine aminotransferase (ALAT)	32	< 37 U/l
Aspartate aminotransferase (ASAT)	28	< 40 U/l

**Fig. 2:**
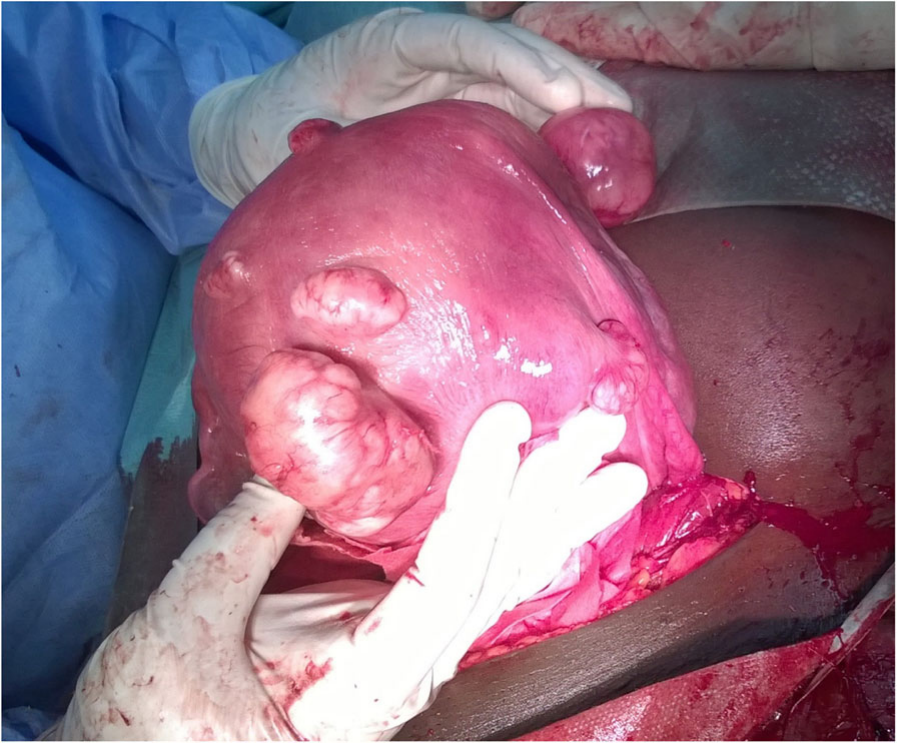
Intraoperative findings; anterior sub-serosal myoma in necrobiosis and posterior sub-serosal myoma

A multidisciplinary team involving general surgeons, obstetricians and anaesthesiologists decided on a two-in one intervention wherby an emergency ceaseraean section with indication severe pre-ecclampsia, and a herniorraphy with indication strangulated umbilical hernia will be carried out within the same operation. Preoperative management consisted of placing two peripheral venous lines of large bore needle with infusion of 1000 ml of normal saline, parenteral administration of an analgesic (paracetamol 1 g), an antihypertensive drug (nicardipine 2 mg bolus) and anticonvulsant (magnesium sulphate 5 g intravenously followed by 4 g intramuscularly in each gluteus muscles). A Pfannenstiel incision performed five hours after admission permitted the extraction of a life female baby who weighed 3300 g at birth with an APGAR score of 8 and 10 at the first and fifth minutes respectively. Intraoperative findings of an anterior and posterior sub-serosal leiomyomas both measuring about 50 mm (Fig. 2); anterior fibroid had an axis pointing to the umbilical ring, with irregular contours and a heterogeneous center, strongly suggestive of aseptic necrobiosis. In addition, the uterus also had several interstitial myomas. The uterine adnexae and the appendix were macroscopically normal. The herniated omentum was not necrosed. No intestines necrosis was observed. Both sub-serosal leiomyomas were surgically excised and sent for histo-pathological evaluation. A separate arciform infra-umbilical incision permitted repair of the umbilical hernia.

**Fig. 3:**
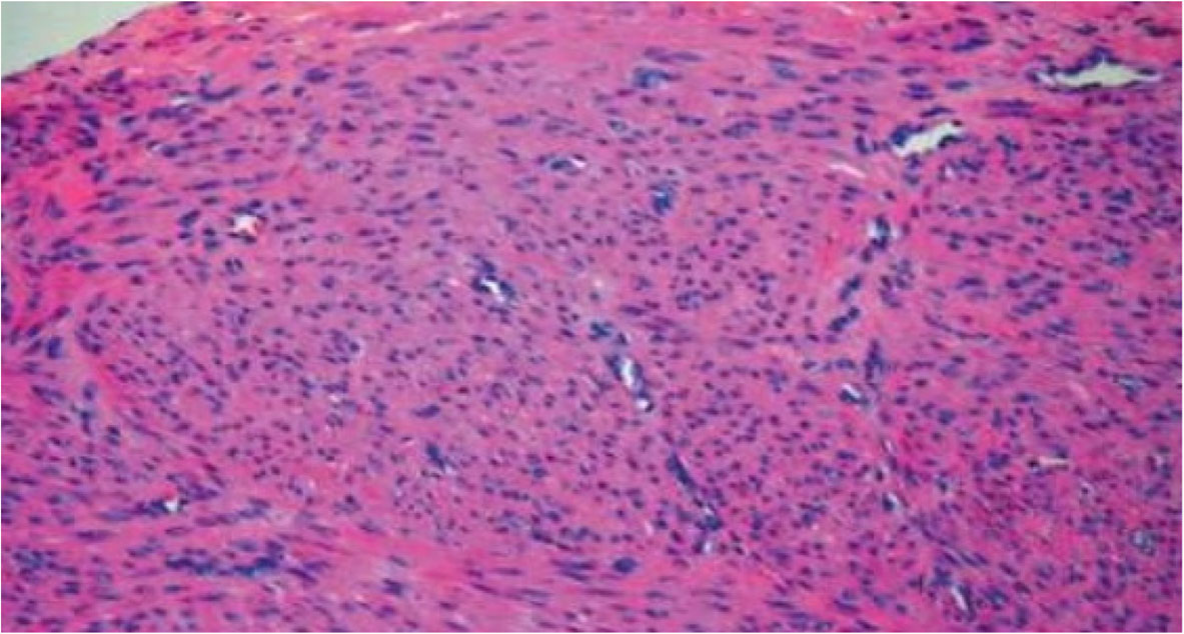
Histopathology of the fibroid showing features of red degeneration

Histopathological analysis of the leiomyoma samples was consistent with red degeneration (aseptic necrobiosis) of the excised uterine fibroid (Fig. 3). The postoperative outcome was uneventful for both the mother and the baby, with the former resuming progressive oral feeding on the first postoperative day. She was discharged five days later in a good clinical condition. Her follow-up at six weeks postoperatively was uneventful.. The six-month postoperative course was also normal.

## Discussion and conclusions

Although no study has shown causality, the evolution of uterine fibroid during pregnancy to aseptic necrobiosis is not uncommon. Aseptic necrobiosis (red degeneration) of a uterine fibroid is defined as a hemorrhagic infarction of a previously hyalinized myoma caused by ischaemic necrosis as the rapid fibroid growth outweighs its blood supply [5]. The incidence of aseptic necrobiosis during pregnancy varies from 1.5% [7] to 28% [8]. Leiomyoma necrobiosis is common among pregnant women compared to non-pregnant women and occurs in two-thirds of cases during the second trimester of pregnancy, although our case occurred in the third trimester of pregnancy [5]. The diagnosis is often made on clinical grounds; a localized, intense and sudden abdominal-pelvic pain, which can be accompanied by hyperthermia inferior to 38,5 °C, nausea and ileus [5]. The investigation of choice is a doppler ultrasound which clearly demonstrates a diminshed or absent blood supply to the myoma [5]. An alternation of cystic and echogenc zones could point to degeneration and infarction [5]. Histopathological demonstration of ischaemic leiomyomatous infarction or necrosis confirms the diagnosis.

Till date, no preventive treatment has been validated for necrobiosis during pregnancy [5]. Only symptomatic treatment is indicated and includes: bed rest, hydration and analgesics (a short bout of non-steriodal anti-inflammatory drugs is generally accepted and well tolerated before 24 weeks of gestation) [5, 9]. These treatments can relieve the patient during the period of acute ischemia. Indications for myomectomy, like all other non-obstetric surgeries during pregnancy, must remain exceptional, reserved for pedunculated sub-serosal fibroids, in torsion or in necrobiosis, resistant to medical treatment [5]. The diagnositic ambiguity in our patient was this particular clinical presentation of the necrobiosis of the fibroma pointing towards the slightly dilated umbilical ring and thus mimicking a strangulated umbilical hernia. The two ultrasound scans performed for diagnostic purposes were rather in favour of a strangulated umbilical hernia with the omental fat as content of the sac. The fibriod in necrobiosis was not visualized despite this clinical suspicion and the history of uterine fibroids. This shows some diagnostic difficulties that can often be encountered in our daily practice.

The main risk of myomectomy during pregnancy is related to haemorrhage [5]. The various cases of myomectomy performed during pregnancy in the literature [9–13], have shown reassuring results, with a normal course of pregnancy and possibilities of vaginal delivery, in future pregnancies. Thereby reinforcing the idea that a surgical alternative is not excluded in the management of leiomyomatous pathology on gravid uteri in selected cases. Generally accepted indications for myomectomy in pregnancy include intractable pain from necrobiosis of a subserosal or pedunculated fibroid and any fibroid lager than 5 cm located on the lower uterine segment [5]. In our case, our patient had pedunculated fibroids which were easily resected. The complexity of the interventions on our patient make it difficult to draw comparisons with previous similar interventions. She had three different surgical procedures in one intervention; a ceaserean section indicated by severe pre-ecclampsia, a myomectomy indicated by an aseptic necrobiososis, and an umbilical herniorraphy indicated by a strangulated umbilical hernia. Prompt intervention by the multidiciplinary team of specialists was key in a favourable outcome for the mother and her baby.

Noteworthy, an important differential diagnosis of acute abdominal pain related to leiomyoma in pregnancy is torsion of a pedunculated subserosal myoma [14, 15]. It is rare for a uterine fibroid to become pedunculated and it is scarcer for pedunculated fibroids to undergo torsion [16, 17]. Till date, the incidence or prevalence of pedunculated subserosal myoma during gestation is unknown. Available data stem from some few published case reports [14, 18–20]. The existence of a pedunculated fibroid is the primary requisite for its torsion [16]. The pedicle of the pedunculated subserous leiomyoma must be long and thin enough to allow excess motility of the fibroid leading to the rotation and subsequent torsion of its stalk [21]. The size of the fibroid determines the reversibility of the torsion process [16]. As such, it is easy to correct torsion spontaneously in twisted pedunculated myomas that are small compared to larger ones [16]. As its stalk rotates, blood vessels supplying the fibroid become strangulated, leading to ischemic or necrotic myomas [16]. Furthermore, a twisted huge pedunculated fibroid can compress surrounding organs like the bowels leading to constipation or ileus during pregnancy and delivery, as well as the puerperal period [22]. The diagnosis of torsion of pedunculated subserous fibroids can be confirmed by ultrasound, computed tomography or magnetic imaging resonance, the most accurate test [21]. However, the diagnosis is often confirmed intraoperatively due to a low pre-operative index of clinical suspicion during pregnancy and limited imaging-based diagnostic characteristics [23], as in our case. Due to the risk of haemorrhagic infarction and subsequent infection [21, 24], the approved management of torsion of uterine fibroids during pregnancy entails emergency myomectomy or artery embolization [16, 25].

In conclusion, this clinical case illustrates the diagnostic difficulties often encountered in the management of aseptic necrobiosis of uterine fibroids during pregnancy. Aseptic necrobiosis of leiomyomas on a gravid uterus is may present as a strangulated umbilical hernia. It is a real differential diagnosis to be evoked by the healthcare providers. Inspite of our hospital infrastructure being deficient in sophisticated medical imaging studies for an accurate pre-operative diagnosis, we have demonstrated that an early multidisciplinary care (obstetricians, surgeons, anesthetists and radiologists) clearly improves the maternal and neonatal outcome. The value of this therapeutic approach is invaluable in a context where maternal and perinatal mortality is of increasing concern.

## Abbreviations

ALAT: Alanine aminotransferase
ASAT: Aspartate aminotransferase
PT: Prothromine Time
TCA: Cephalokaoline time
WBC: White blood cell count

## References

[1] Day Baird D, Dunson DB, Hill MC (2003). High cumulative incidence of uterine leiomyoma in black and white women: ultrasound evidence. Am J Obstet Gynecol.

[2] Cramer SF, Patel A (1990). The frequency of uterine leiomyomas. Am J Clin Pathol.

[3] Poovathi M, Maternal RR (2016). Fetal outcome in Pregnancy with fibroids: a prospective study. Int J Sci Stud.

[4] Rongières C (1999). Épidémiologie du fibrome utérin: facteurs de risque et fréquence. Impact en santé publique. J Gynecol Obstet Biol Reprod Paris.

[5] Lee HJ, Norwitw ER, Shaw J (2010). Contemporary Management of Fibroids in pregnancy. Rev Obstet Gynecol.

[6] Dohbit JS, Meka E, Tochie JN, Kamla I, Mwadjie D, Foumane P (2017). A case report of bicornis bicollis uterus with unilateral cervical atresia: an unusual aetiology of chronic debilitating pelvic pain in a Cameroonian teenager. BMC Womens Health.

[7] Strobelt N, Ghidini A, Cavallone M, Pensabene I, Ceruti P, Vergani P (1994). Natural history of uterine leiomyomas in pregnancy. J Ultrasound Med.

[8] Dilucca D. Fibrome et grossesse. À propos de 476 cas. Thèse, Paris. 1981.

[9] Sentilhes L, Sergent F, Verspyck E, Gravier A, Roman H, Marpeau L (2003). Laparoscopic myomectomy during pregnancy resulting in septic necrosis of the myometrium. BJOG.

[10] Bhatla N, Dash BB, Kriplani A, Agarwal N (2009). Myomectomy during pregnancy: a feasible option. J Obstet Gynaecol Res.

[11] De Carolis S, Fatigante G, Ferrazzani S (2001). Uterine myomectomy in pregnant women. Fetal Diagn Ther.

[12] Celik C, Acar A, Ciçek N (2002). Can myomectomy be performed during pregnancy?. Gynecol Obstet Investig.

[13] Wittich AC, Salminen ER, Yancey MK, Markenson GR (2000). Myomectomy during early pregnancy. Mil Med.

[14] Gaym A, Tilahun S (2007). Torsion of pedunculated subserous myoma--a rare cause of acute abdomen. Ethiop Med J.

[15] Foissac R, Sautot-Vial N, Birtwisle L, Bernard JL, Fontaine A, Boujenah S, Benchimol D, Bereder JM (2011). Torsion of a huge pedunculated uterine leiomyoma. Am J Surg.

[16] Tsai YJ, Yeat SK, Jeng CJ, Chen SC (2006). Torsion of a uterine leiomyoma. Taiwanese J Obstet Gynecol.

[17] Gupta S, Manyonda IT (2009). Acute complications of fibroids. Best Pract Res Clin Obstet Gynaecol.

[18] Basso A, Catalano MR, Loverro G, Nocera S, Di Naro E, Loverro M, Natrella M, Mastrolia SA (2017). Uterine fibroid torsion during pregnancy: a case of Laparotomic myomectomy at 18 weeks’ gestation with systematic review of the literature. Case Rep Obstet Gynecol.

[19] Kim HG, Song YG, Na YJ, Choi OH (2013). A case of torsion of a subserosal leiomyoma. J Menopausal Med.

[20] Kakou C, Kasse R, Garba I, Gondo D, Boni S (2017). Torsion of uterine fibroid: a rare cause of acute pelvic pain: about one case. Gynecol Obstet Case Rep.

[21] Lai YL, Chen YL, Chen CA, Cheng WF (2018). Torsion of pedunculated subserous uterine leiomyoma: a rare complication of a common disease. Taiwanese J Obstet Gynecol.

[22] Mickel I, Bollmann R, Chaoui R, Lau HU (1995). Torsion of the myoma pedicle as a rare cause of ileus in puerperium. Geburtshilfe Frauenheilkd.

[23] Marcotte-Bloch C, Novellas S, Buratti MS, Caramella T, Chevallier P, Bruneton JN (2007). Torsion of a uterine leiomyoma: MRI features. Clin Imaging.

[24] Roy C, Bierry G, Ghali SE, Buy X, Rossini A (2005). Acute torsion of uterine leiomyoma: CT features. Abdom Imaging.

[25] Katsumori T, Akazawa K, Mihara T (2005). Uterine artery embolization for pedunculated subserosal fibroids. AJR Am J Roentgenol.

